# A Cloud-Computing-Based Portable Networked Ground Station System for Microsatellites

**DOI:** 10.3390/s22093569

**Published:** 2022-05-07

**Authors:** Yifei Jiang, Shufan Wu, Qiankun Mo, Wenzheng Liu, Xiao Wei

**Affiliations:** 1 School of Aeronautics and Astronautics, Shanghai Jiao Tong University, Shanghai 200240, China; jamesjiang@sjtu.edu.cn (Y.J.); moqk@sjtu.edu.cn (Q.M.); 2 Shanghai Aerospace Systems Engineering Institute, Shanghai 201101, China; roadking@163.com (W.L.); weixiao805@163.com (X.W.)

**Keywords:** microsatellites, cloud computing, ground station

## Abstract

Microsatellites have attracted a large number of scholars and engineers because of their portability and distribution characteristics. The ground station suitable for microsatellite service has become an important research topic. In this paper, we propose a networked ground station and verify it on our own microsatellite. The specific networked ground station system consists of multiple ground nodes. They can work together to complete data transmission tasks with higher efficiency. After describing our microsatellite project, a reasonable distribution of ground nodes is given. A cloud computing model is used to realize the coordination of multiple ground nodes. An adaptive communication system between satellites and ground stations is used to increase link efficiency. Extensive on-orbit experiments were used to validate our design. The experimental results show that our networked ground station has excellent performance in data transmission capability. Finally, the specific cloud-computing-based ground station network successfully completes our satellite mission.

## 1. Introduction

With the rapid development of satellite technology, a large number of new topics are proposed every year [1,2,3]. Among the many research directions, the topic of microsatellites has attracted the attention of a large number of scholars and engineers. Its advantages include miniaturization, low cost and short development cycle. In order to realize a superior microsatellite system, a high-performance data transmission system is essential.

In addition to the design of satellites, the ground station is an indispensable component of satellite systems [4]. The harsh space environment, low power ability and small size of antennas make the communication ability of microsatellites poor [5]. Traditional ground stations cannot meet the stringent requirements of microsatellites

For microsatellites, a sun-synchronous orbit (SSO) is utilized for more energy from the sun. Unfortunately, the distance from the SSO to a fixed ground node could change three times with the movement of satellites [6], which will affect the received signal strength. The traditional solution for this inherent flaw is to reserve more margin in the link budget, but it is a luxury and sometimes impossible for microsatellites. For low transmission delay, a low altitude is selected by the SSO. However, on the other hand, the lower the orbit altitude, the shorter the link duration. This drawback makes information throughput insufficient for telemeter (TM) and telecommand (TC).

Recently, there have been many studies on satellite ground stations. Refs. [7,8,9] propose some hardware designs of ground station equipment are introduced. Novel antenna and GPU boards are found to improve ground station performance at the cost of resource consumption. In [10], a throughput-based transmission plan is introduced. In [11], Liashkevich designs an X-band university ground station with the SDR platform and a big dish antenna to track NOAA-20, Terra and another low-orbit satellite. However, it is found that short link duration worsens the performance of the system. This design only contains the function of a single receiver without any cooperation. Kleinschrodt [12] analyzes the impact on in-orbit ground station communication. Static performance and dynamic performance are introduced by analysis of the sensitivity, frequency correction and tracking accuracy of the ground station. However, the important parameters are fixed in the design phase and cannot be changed. The flexibility of the communication system is not considered in the design. Bauomy [13] introduces an SDR simulation for ground stations and CubeSats. The aim of this paper is to assist in the design of communication systems. Wei receives [14] TM signals from lunar microsatellites through an SDR-based ground station. Although this design is excellent, they did not take into account the multiple access of the network. Sara introduces a single-satellite and multiground optimization schedule in [15]. This strategy excels at ground station resource arrangment. It is useful in the design of complex satellite ground systems. However, such complex algorithms are not suitable for microsatellites and portable ground stations. A community-driven approach [16] is used to solve the access problem between satellites and ground stations. A cloud database is used to store the information received from satellites. An amateur ground station is designed in [17]. It satisfies the miniaturization of portable requirements. However, the receiver performance is sacrificed for size. A small satellite ground station is introduced in [18], used for VZLUSAT-1. This article proposes a ground station system that can serve multiple small satellites simultaneously. However, the link duration of each satellite is limited. Ref. [19] proposes ground station networks used in the processing of GNSS signals. Massive raw data are stored in the ground station networks. However, this system cannot increase the efficiency of data transmission through networks. Ref. [20] proposes a ground station link plan used for data transmission in GPS constellations. Massive data transmission is realized by this strategy. The average throughput verifies the design. However, this system cannot satisfy the flexibility requirements of communication satellites.

SSS-2A is a microsatellite developed by Shanghai Jiao Tong University. It belongs to the Student Small Satellite (SSS) project organized by the Asia-Pacific Space Cooperation Organization (APSCO). SSS-2A was launched on 14 October 2021. As the developer of SSS-2A, our mission is not only the design of microsatellite but also telemeter (TM), telecommand (TC) and data transmission (DT). Due to the limitations of microsatellites such as low transmit power, unstable attitude control and random working state, all reception performance needs to be guaranteed by the excellent performance of the ground station system.

Although there is a lot of research on satellite ground stations, existing research cannot meet the multiple requirements at the same time. In our project, a system is required to satisfy both massive data transmission, high communication efficiency, miniaturization and low power consumption. Short link duration and dynamic link attenuation are the two most serious problems. The former will reduce the amount of received data. The latter will greatly reduce the energy efficiency of microsatellites. Miniaturization and low consumption are also important for performance. Unfortunately, existing research or designs cannot satisfy the above requirements simultaneously.

Based on the above analysis, we propose a networked ground station system for the SSS-2A mission. A cloud server, adaptive communication and SDR structure all play an important role in the system. A cloud server is used to solve the short link duration problem. An adaptive communication module excels at the stability of link attenuation fluctuations. The software-defined radio (SDR) structure can realize miniaturization and low power consumption. On-orbit data reception is implemented as verification.

## 2. Related Work

There are many related satellite ground station studies. Ref. [16] proposes a community-driven approach used to solve the access problem between satellites and ground stations. Although this approach is based on cloud servers, it is unable to simultaneously control multiple ground stations. The cloud server only acts as a database. The link duration is not enough to transmit massive data. Refs. [21,22] proposes a networked ground station which is introduced to realize data transmission. However, the management of data does not include task assignment and resource management. This defect greatly reduces the efficiency of data reception. Ref. [23] proposes a ground station assignment strategy which is used to increase communication efficiency. However, a lot of computation increases the size and power consumption of the ground station, which cannot satisfy the miniaturization requirements. Ref. [24] proposes an adaptive feeder link which is used to realize the adaptivity of communication systems. However, this optical system cannot be used in our satellites. Refs. [25,26,27,28] propose many adaptive systems for mobile communication, intervehicle communication and laser communication. Due to the dynamic changes in satellite communication, they cannot be used directly in satellite-to-ground communication systems. Refs. [29,30,31] propose many cloud computing systems for satellite application. They contain a large number of data interaction strategies. However, they cannot adequately balance performance with efficiency.

To further understand our requirements, the ten most relevant studies were compared in detail. As shown in Table 1, there are four important parameters defined as judging criteria. Most existing research only meets one requirement. This will greatly hinder the execution of our SSS-2A mission.

According to the characteristics and requirements of SSS-2A, a ground station system should be able to solve multiple problems at the same time. Short link duration, link attenuation fluctuations, miniaturization and low power consumption are the main requirements. Unfortunately, no existing research has been able to meet these requirements simultaneously.

The main innovations of this paper are as follows. In order to improve the link duration time, a virtual link is constructed by combining multiple ground nodes. This function can only be realized based on cloud computing. In order to increase communication efficiency, adaptive communication based on a feedback system is constructed. To meet the requirements of miniaturization and low power consumption, a ground station based on the SDR architecture is designed. This system dynamically controls the power supply of all devices according to cloud commands. This measure greatly reduces power consumption. An on-orbit verification experiment is more convincing than other simulations and tests.

## 3. Mission Description

The SSS project organized by APSCO contains three Student Small Satellite subprojects: SSS-1, SSS-1A and SSS-2A. SSS-1 is a small satellite weighing about 50 kg, carrying an ADS-B receiver as its main payload. SSS-1A and SSS-2A are microsatellites with 3U volume. SSS-2A, equipped with an AIS receiver and intersatellite communication as payloads, was developed by Shanghai Jiao Tong University (SJTU). The UHF and VHF bands are selected as the main channels for telemeter (TM), telecommand (TC) and data transmission (DT). In addition to the development of microsatellites, the ground station system is also developed by ourselves.

### 3.1. Orbit Design

A sun-synchronous orbit (SSO), also called heliosynchronous orbit, is the near-polar orbit around a planet. In SSO, the satellite passes over any given point of the planet’s surface at the same local mean solar time. It always maintains the same relationship with the sun. This advantage is beneficial to small satellites because of their low power consumption.

**Figure 1:**
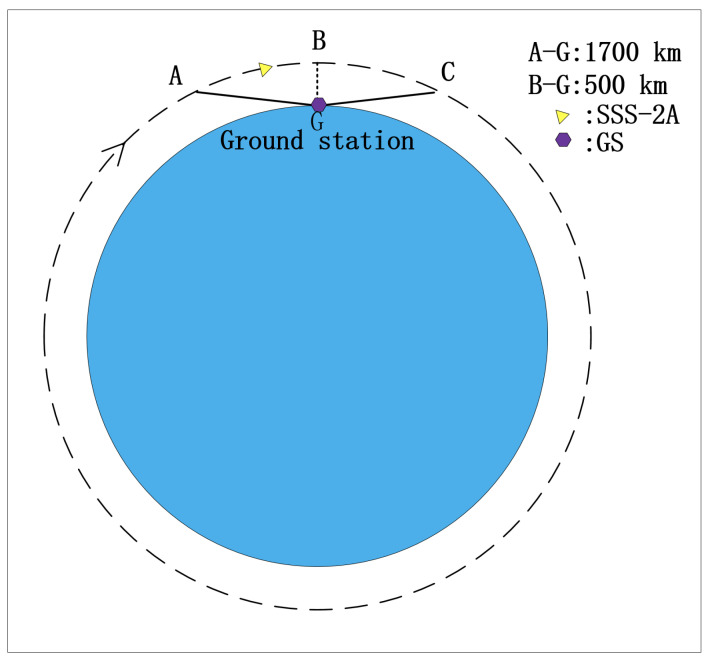
Sun-synchronous orbit (SSO) feature.

In the SSS project, a 500 km SSO is defined as the operating orbit. The distance from the satellite to a given ground node changes from 500 km to 1700 km, and the pitch angle changes from 90° to 10°. The relative velocity changes from 0 to 7 km/s. As shown in Figure 1, the satellite moves clockwise around Earth, from Points A to B and C. The space attenuation of the link changes from −130 dB to −160 dB.

For a definite orbit, the link attenuation can be calculated in detail. The minimum received power is −160 dBm. The maximum doppler offset is 10 KHz for the UHF band and 6 KHz for the VHF band. The results of these calculations are important boundary conditions for the design of ground stations.

### 3.2. TM, TC and DT Analysis

**Figure 2:**
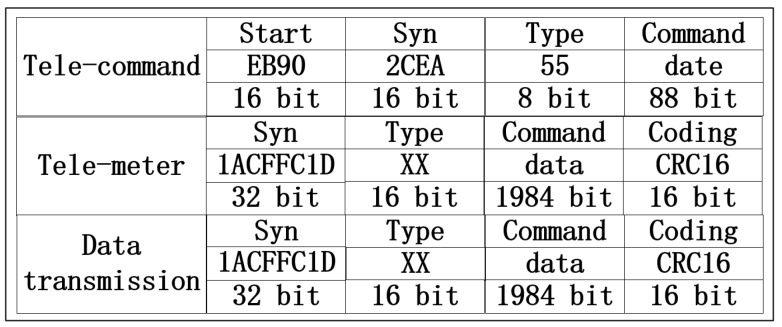
SSS-2A data packets.

For the requirements of SSS-2A, there are three different data packets in our communication system: telecommand (TC), telemeter (TM) and data transmission (DT). The TC packet contains 128 bits and can be divided into four components. The TM and DT packets contain 2048 bits and can also be divided into four components. The details of the three different packets are shown in Figure 2.

**Figure 3:**
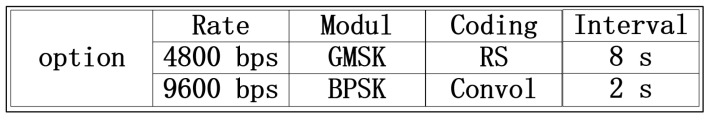
Communication mode option.

According to the agreements, there are two different modulation methods that can back each other up, namely GMSK and BPSK. Two different data rates, 9.6 kbps and 4.8 kbps, are available. Two different coding types, RS coding and convolution coding, are options for our design. As shown in Figure 3, the transmit interval between two packets is also optional.

TM packets should be responsible for the transfer of the data from the integrated electronic subsystem, attitude and orbit subsystem and power subsystem. Since a large number of data packets will be automatically generated by the satellite, it is very important to efficiently arrange the data transmission. TC packets should be responsible for the transfer of the data from the ground command center. DT packets should be responsible for the transfer of the data from payloads.

According to ITU standards, the VHF band is selected as the TC channel, and the UHF band is selected as the TM and DT channel. These bands are also amateur radio frequency bands; thus, an amateur license is also a legal permit for our ground stations.

### 3.3. Microsatellite Communication System Design

**Figure 4:**
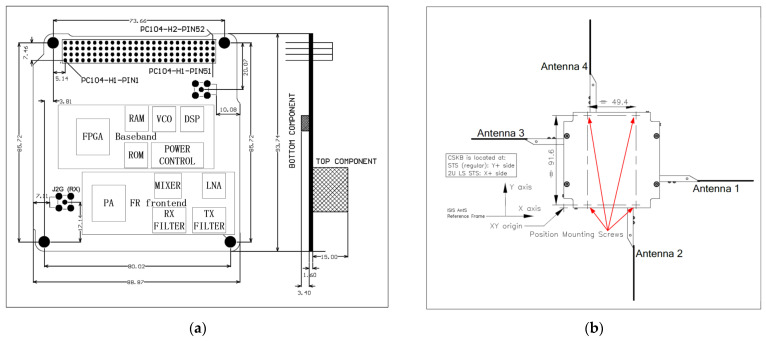
(**a**) UVH transceiver design; (**b**) UVH antenna design.

Since SSS-2A is a microsatellite, the size of the motherboards of all equipment must meet the conditions. As shown in Figure 4, the envelopes of the UVH transceiver and UVH antenna are compatible with the PC/104 mechanical specifications.

The UVH transceiver consists of digital baseband components and RF frontend components. It can also transmit and receive signals. In our mission, the downlink frequency for TM and DT is 435.775 MHz, and the uplink frequency for TC is 145.985 MHz. The internal power amplifier (PA) outputs a 0.5 W RF signal to the antenna. The average gain of the specific antenna is −3 dB; thus, the equivalent isotropically radiated power (EIRP) can be defined as 24 dBm.

For miniaturization, a set of dipole antennas is placed on the top of the satellite. Two orthogonal linear polarization characteristics can increase the isolation of transceivers and also increase the difficulty of ground equipment.

## 4. Ground Station Network

Short link duration is the fatal flaw of LEO microsatellites. Fortunately, this shortcoming can be compensated for with a collaboration method. Multiple nodes work together to serve the same satellite to increase the total link duration.

Before the design of ground stations, suitable locations should be selected. Since urban noise, building occlusion and environmental factors can all affect the performance of the ground stations, the location selection of the ground stations is particularly important. As an important test and experiment, sparsely populated places are more suitable for building ground stations. However, the specific location distribution of the ground stations also depends on the mission requirements.

### Network Topology

**Figure 5:**
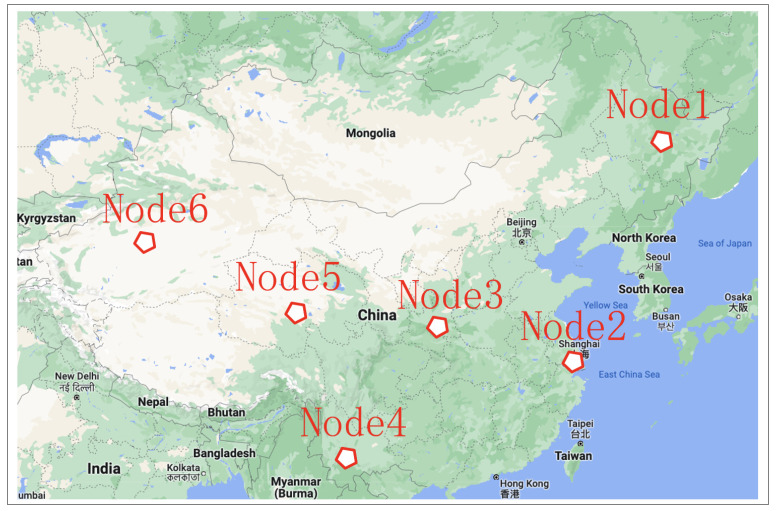
Ground station network topology.

For excellent payload performance, a large amount of data needs to be transmitted from the satellite. To compensate for the short link duration caused by orbit characteristics, multiple ground nodes should be scattered throughout the country. They should be spread evenly across longitudes and latitudes. Based on the actual situation in China, six regional ground stations are selected. They are No. 1, Haerbin (128°4′, 45°3′); No. 2, Shanghai (121°3′, 31°0′); No. 3, Xi’an (108°4′, 33°8′); No. 4, Yunnan (103°4′, 25°3′); No. 5, Qinghai (95°0′, 36°2′); and No. 6, Xinjiang (80°6′, 39°4′), as shown in Figure 5.

The coverage of the selected six ground nodes contains most of the country’s land area. The specific topology can guarantee more connection opportunities. In order to verify the topology, we simulate the link access between the satellite and ground nodes with the commercial software STK. As shown in Table 2, the proposed topology obtains 18 connection opportunities in 24 h. Each link has a different duration, pitch angle and distance. For instance, Links 5, 8, 12 and 15–17 have a duration long enough to transmit large data packets. Different tasks should be assigned to different links.

For some links, such as Links 4 and 5, their overlapping parts need to be further processed for higher efficiency. Some adjacent connections can be constructed as a continuous link. This virtual link consists of links of multiple different ground nodes. By these methods, it is possible to artificially establish longer durations.

**Figure 6:**
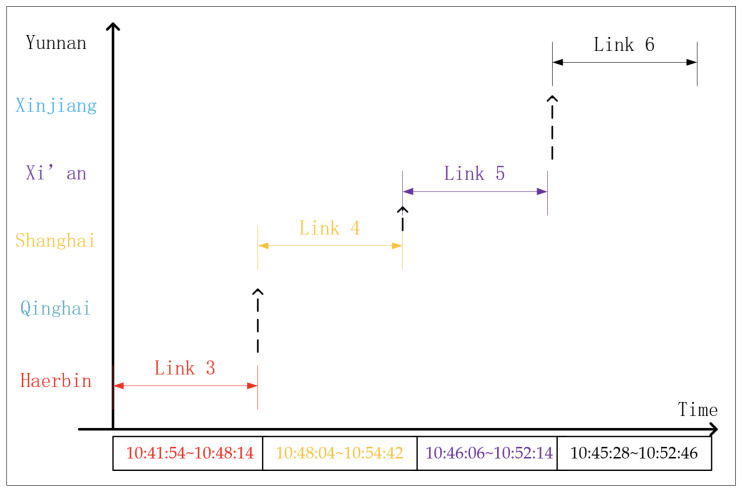
Result of simulation by STK.

As described in Table 2 and shown in Figure 6, Links 3–6 overlap each other. When selecting the link from Links 3 to 4 and Links 5 to 6 in turn, a virtual continuous link duration from 10:41:54 to 10:54:42 can be established. This virtual link duration lasts 720 s, which is far longer than any single link duration. Similarly, Links 12, 13 and 15 can establish another virtual link duration.

Through collaboration of the virtual link and multiple ground stations, the duration of the new ground link is greatly increased. Compared with the individual mode, collaboration can save a lot of overhand between satellites and ground stations.

In this section, we proposed a ground station topology with six ground nodes and simulate it with STK. This appropriate topology can improve the link duration and coverage of ground stations. However, cooperative control, establishment of virtual links and reconfiguration of overlapping links all require a large amount of computation. This computing power greatly increases the burden on the ground station. Fortunately, cloud computing technology can solve this problem.

## 5. Cloud Computing

For excellent satellite-to-ground data transmission capability, multiple ground stations are required to collaborate. It is also an important task to collect received data packets scattered at various ground stations. Predicting the link access according to the TLE of satellites and allocating access tasks to each ground station will also consume a lot of computing power. These main functions all rely on a series of self-developed algorithms. They will be running on cloud servers.

### 5.1. Massive Data Management

In each connection opportunity, hundreds of TM and DT packets can be received from SSS-2A, and dozens of TC packets can also be transmitted. A large amount of data processing is complex. The processing of virtual links is more complicated.

**Figure 7:**
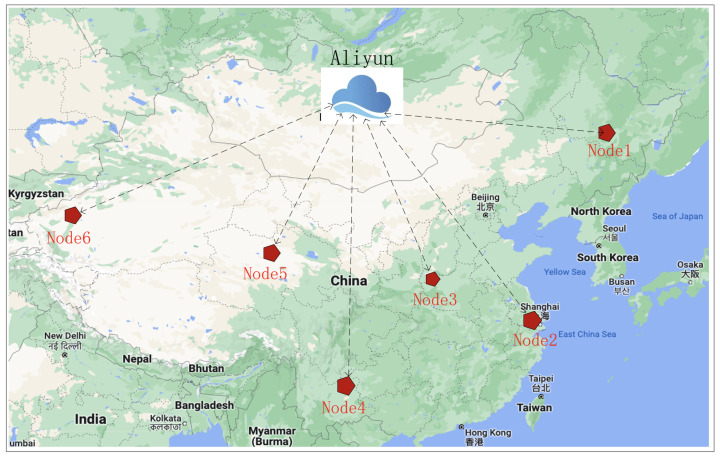
Cloud computing structure.

As shown in Figure 7, through the cloud server, six scattered ground stations can be managed uniformly. The cloud data center will collect and store the data packets in real time. Similarly, the TC packet will also be distributed to the corresponding ground station. In the cloud server, there are three different databases for storing and processing different data packets. These data can be visited by an authorized customer via a network interface.

#### 5.1.1. Upload Data Flow

**Figure 8:**
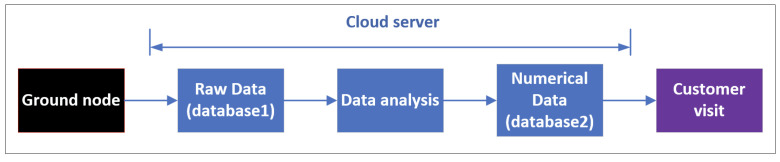
TM and DT data flow.

Once a TM or DT packet is received successfully, a set of raw data with 256 bytes is stored in the ground station. It will be uploaded to the cloud data center by the terrestrial network immediately. As shown in Figure 8, the cloud server will store raw data in database1. The raw data are unable to express the real information of satellites or payloads. A data analysis block is designed to translate raw data into numerical data, which is identifiable by the customer. The numerical data are stored in database 2. Any authorized user can access the desired information through the UI interface.

There is a feedback mechanism to ensure the reliability of data collection. The ground station will keep the uploaded data until it receives an acknowledgment (ACK) from the cloud server.

#### 5.1.2. Download Data Flow

**Figure 9:**
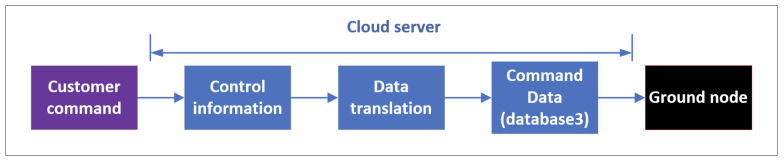
TC data flow.

The download flow is responsible for TC packets and satellite update packets. As shown in Figure 9, an authorized user can transmit data packets to specific ground stations. Similarly, TC packets should also be converted.

Any data processed by the cloud data center will be stored for a long time. In the case of negligent operation, any transmitted data packets will be recorded in a log. For excellent performance, different software will be applied in our cloud data center, as shown in Table 3. For miniaturization and low power consumption, all computer power is borne by the cloud server. In order to increase the scalability of the server, a Linux system is used as the underlying driver.

The choice of cloud server is to balance the contradiction between large computing power and low power consumption. In order to increase the link duration, it is necessary to construct a virtual link by a large amount of computation. However, the low power consumption requirement makes the ground station unable to bear such a large computational burden. Remote computing by cloud server is the best solution.

### 5.2. Access Link Assignments

**Figure 10:**
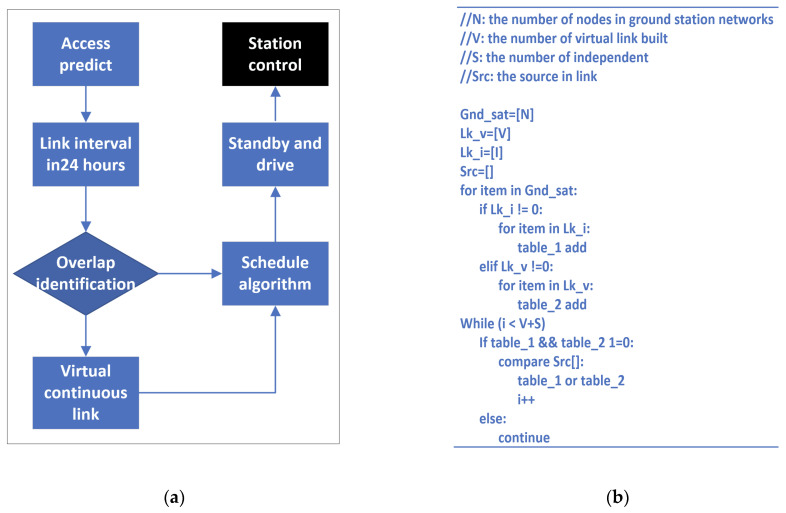
(**a**) Assignment flow; (**b**) pseudocode of schedule algorithm.

To improve the efficiency of the ground station in the time resource and frequency resource, the time division multiple access (TDMA) strategy is preferred. In this strategy, we establish a schedule to manage connections between the satellites and multiple ground stations. As shown in Figure 10a, there are six steps in this strategy.

- Access prediction: Calculate the connectable period between SSS-2A and each ground station with the LTE of SSS-2A. This calculation can be accomplished with the “ephem” library in python.
- Overlap identification: As simulated in Section 4, many link durations may overlap each other. Identifying overlaps can increase assignment efficiency.
- Construction of virtual link: For some special link durations, they can be combined into a virtual continuous link.
- Schedule algorithm: Based on the above classification and construction, an efficient algorithm is the core of the entire strategy.
- Driving mechanism: A lot of equipment in the ground stations needs to be warmed up. The driving mechanism can control the operation of the equipment according to the schedule. As shown in Table 4, the warm-up times of different devices are shown.

For the schedule algorithm, there are many algorithm structures available. Simplicity, stability and high efficiency are the most important. A Bellman–Ford algorithm is competent in this application because of high reliability. The pseudocode is shown in Figure 10b.

The implementation of virtual links relies on a high-performance schedule. According to the assignments, the ground station system can maximize the data transceiver capability. The establishment of virtual links is realized with the help of cloud computing.

Once the schedule has been established, the controller of the ground stations will run automatically. All received packets will be uploaded to the cloud data center. The TC packets will also be transmitted as required. These collaborative operations not only improve the link durations but also make adaptive communication possible.

## 6. Adaptive Communication

An actual communication system has to compromise between performance and resource consumption. For microsatellites, the restricted power and frequency resource affect the throughput seriously. Dynamically adjusting the communication parameters can improve system efficiency. As shown in Figure 3, some important parameters are optional for synchronization, owing to the flexibility of the software-defined radio (SDR) structures. Different choices of those parameters make the system exhibit different performances. It can be seen from some contrasts that the 9600 bps data rate uses double the bandwidth than the 4800 bps data rate in the same situation. BPSK has better noise immunity than GMSK. However, the former has to contain equalizer and timing recovery modules. There are two different transmit intervals. We define the long interval as the slow packet mode and the short interval as the quick packet mode. The slow mode is more suitable than the quick mode in spare time. The choice of different coding methods is determined by the signal-to-noise ratio (SNR).

**Figure 11:**
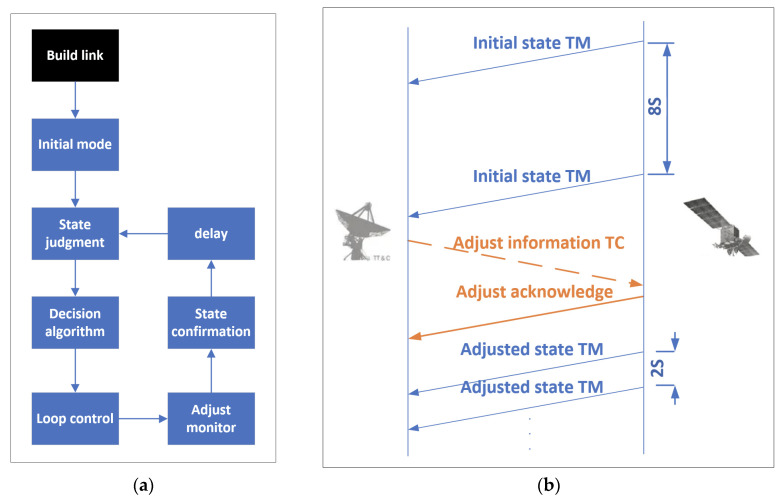
(**a**) Adaptive adjustment flow chart; (**b**) self-loop diagram.

A connection is established with the initial state. The communication parameters will be adjusted by a self-loop between the satellite and ground station. As shown in Figure 11, when a satellite enters the coverage of the ground station, it will build a basic link as the initial mode. At the beginning, the most reliable communication parameters will be chosen. After the initial mode is stable, an adjust information TC packet and an adjust acknowledge TM packet are applied to accomplish the self-loop. Finally, the adjusted-state TM packets are transmitted according to the new communication parameters. In order to match the dynamic changes of the channel, such a self-loop will be repeated periodically during the entire connection period.

Specific adaptive communications are accomplished by the interaction between the TM packets and TC packets. However, a complex decision algorithm is the core of an adaptive system. In this algorithm, the decision will be made according to distance, power resource, azimuth and pitch angle. The optimal solution is the key information of adaptation. For miniaturization and low power consumption, this algorithm will be embedded in the Aliyun Cloud server.

A satellite ground adaptive system can greatly improve communication efficiency. Adaptation must be based on the exchange of information. A feedback system between the satellite and ground station can improve the efficiency of the satellite ground communication system.

## 7. Design of Portable Ground Station

**Figure 12:**
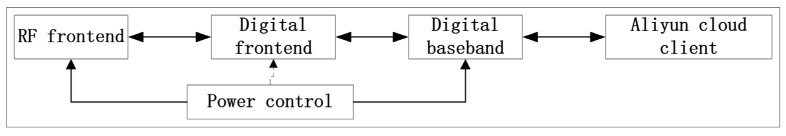
Ground station receiver design.

As described in Section 5, the networked ground station system consists of several independent ground stations. As shown in Figure 12, the unified design of the ground stations contains a digital frontend module, digital baseband module, radio frequency frontend module and power control module. Each module implements different functions, which work together to form a complete system.

### 7.1. Digital Frontend Module

The conversion between digital signal and analog signal is an important process in the communication system. As shown in Figure 12, a digital frontend module is responsible for DAC/ADC, digital filter, digital resampling and another conversion function. The software-defined radio (SDR) is a radio communication system. The hardware of the SDR is replaced by an embedded system. For a portable design, the SDR is our best choice.

The USRP B210 SDR provides a fully integrated, single-board, universal software radio peripheral platform with continuous frequency coverage from 70 MHz to 6 GHz. The integrated RF frontend on the USRP B210 is designed with the new Analog Devices AD9361, a single-chip direct-conversion transceiver, capable of streaming up to 56 MHz of real-time RF bandwidth.

**Figure 13:**
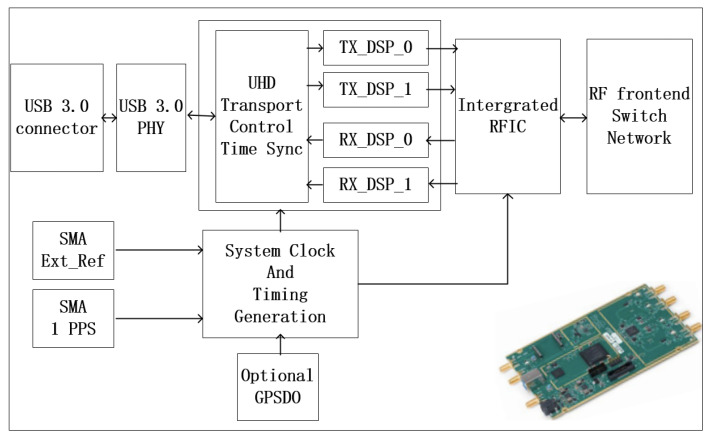
USRP B210 structure diagram.

As shown in Figure 13, B210 contains two individual channels. It can satisfy both TC and TM functions simultaneously. A single B210 meets the digital conversion requirements of the entire ground station. However, B210 is only the hardware part of the SDR platform, and the driver software should be embedded in the digital baseband module.

### 7.2. Digital Baseband Module

The core functions of a ground station are modulation/demodulation, coding/decoding, frequency capture, time correction and data forwarding. In SDR architectures, such core functions should be implemented in a microcomputer such as a PC, SoC and ARM. In our design, a Raspberry Pi 4 is chosen to undertake the digital baseband functions.

As shown in Table 5, there are six different parts in the digital baseband module. They undertake different functions. In particular, the demodulation blocks of BPSK and GMSK are specially designed by ourselves for better performance.

**Figure 14:**
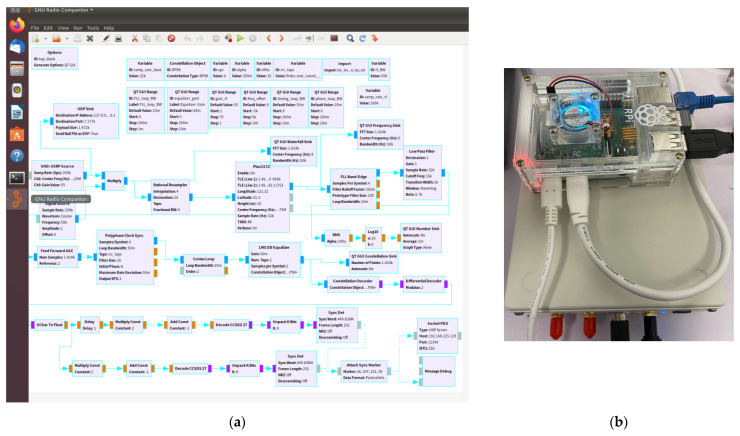
(**a**) Demodulation flow; (**b**) digital frontend and digital baseband modules.

The basic flows of demodulation and decoding are shown in Figure 14a, two different modulation types will be selected according to the control from the cloud computing script. Similarly, two different coding types will also be selected. The adaptive communication blocks are embedded in the demodulation modules.

As shown in Figure 14b, the B210 SDR platform and Raspberry Pi 4 are compact enough to be fixed in a mobile terminal. The power consumption is less than 5 W, which can be supplied by a power bank.

### 7.3. Radio Frequency Frontend Module

**Figure 15:**
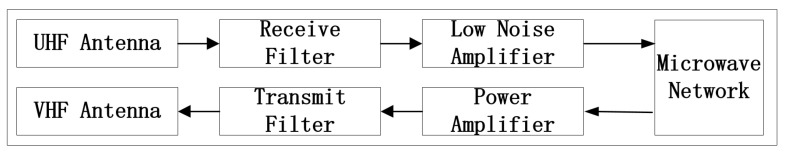
RF frontend flow.

The radio frequency (RF) frontend is an important component in wireless communication systems. It contains antennas, filters, amplifiers, mixers and microwave networks. As shown in Figure 15, UHF is the downlink frequency, and VHF is the uplink frequency in our system. Each component in downlink and uplink plays a different role.

#### 7.3.1. Antenna Design

**Figure 16:**
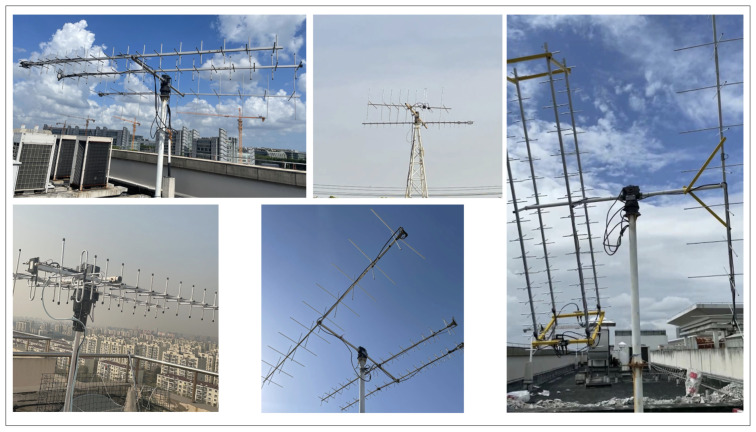
Ground station network antenna design.

A pair of linear polarized dipole antennas are mounted on the top of our microsatellite. To compensate for the random attitude and low gain, a circularly polarized Yagi antenna is our best choice in the ground station. Each ground station contains a VHF circularly polarized Yagi antenna and a UHF circularly polarized Yagi antenna array. To track the satellites, a Yaesu G-5500 Azimuth-Elevation rotator system is applied. The G-5500 can make the antennas point precisely at the moving satellite. The cloud server calculates the pointing parameter through TLE and transmits it to the ground station for execution. To track our satellite continuously, multiple antennas should work together. As shown in Figure 16, several different antennas are distributed all over the country.

#### 7.3.2. Filter and Amplifier Design

The receive filter and transmit filter have different requirements in design. The former requires low insertion loss and high out-of-band rejection; the latter requires high power tolerance. Surface acoustic wave (SAW) devices are selected as the receive filter and cavity devices as the transmit filter.

As shown in Table 6, LNA requires a low-noise figure, and PA requires high output power. Because of the different voltages utilized by LNA and PA, a more efficient power control module is necessary.

### 7.4. Power Control Module

**Figure 17:**
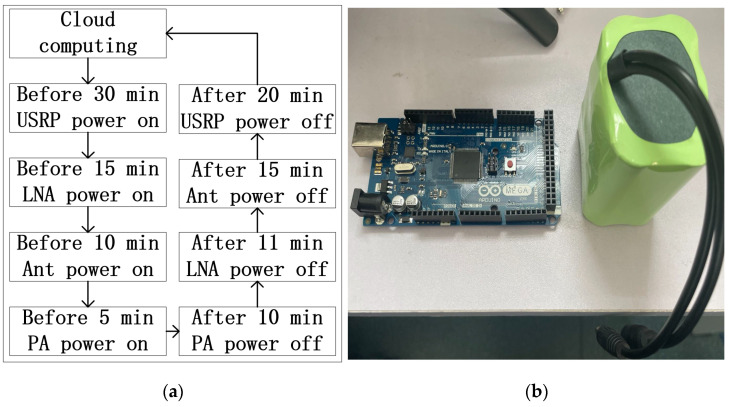
(**a**) Power control flow; (**b**) power control devices.

In order to increase energy efficiency, the power supply of the ground station should be controlled according to the cloud computing instructions. As shown in Figure 17a, when a satellite approaches the ground station, some equipment of the ground station will be powered gradually. Except for some networked equipment, other unrelated devices will be powered off during idle time. This strategy will greatly reduce unnecessary power consumption.

Power control commands are provided by cloud server and transmitted to Raspberry Pi 4 directly. As shown in Figure 17b, an Arduino microcontroller is used as the hardware of the power control module. Using the program developed by ourselves, various power control functions are possible in the ground station.

This module can greatly reduce the power consumption of the ground station. Complex processing is embedded in the cloud servers.

In this section, we introduced four modules, namely the digital frontend module, digital baseband module, RF front end module and power control module. The ground station is designed to meet the requirements of small phones and low power consumption.

## 8. Experiments and Verifications

To verify the credibility of the design, extensive experiments and data analysis are presented. During development, extensive simulation was used to aid the design. However, any simulations have uncertainty. On-orbit verification is the most convincing. In this paper, an on-orbit data service is implemented to verify the system.

The SSS-2A microsatellite was launched from the Taiyuan site at 19:05 Beijing time and successfully entered the predetermined orbit. According to calculations, the first entry into Chinese territory will be at 20:35 Beijing time, and a connection with the Xinjiang ground station will be established. The success of this connection determines the success of the entire mission. After the Xinjiang ground station, several connections can be established with Haerbin, Shanghai, Xi’an, Yunnan and Qinghai in tune. The performance of the networked ground stations can be verified in subsequent connections.

### 8.1. Ground Station Experiments

Before SSS-2A enters the coverage of the ground station, all equipment will power on and warm up. According to our design, when the pitch angle is greater than 10 degrees, the ground station will be operated in the initial state. Once a connection is successfully established, the adaptive system starts.

**Figure 18:**
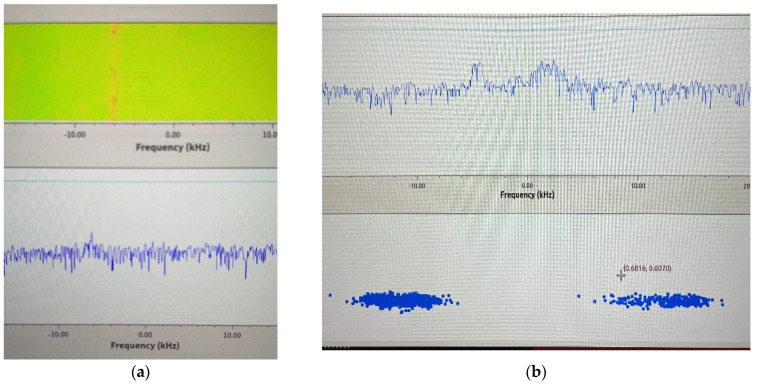
(**a**) GMSK signal spectrum; (**b**) BPSK signal spectrum.

As shown in Figure 18, the SDR platform receives the signal and displays it in the form of a spectrum. Figure 18a,b shows the received GMSK and BPSK signals, respectively. The former is the initial state of the system, and the latter is the result of adaptive adjustment.

**Figure 19:**
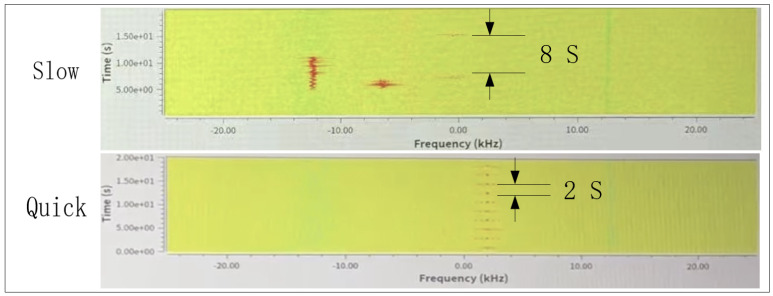
Short and long intervals schematics diagram.

As shown in Figure 19, the long and short intervals correspond to slow packets and quick packets, respectively. At the beginning, TM packets are transmitted in the initial state with a cycle of 8 s and switched to a cycle of 2 s according to the command.

The switching of coding method and data rate will be reflected in subsequent data analysis. Once the signal demodulation is successful, the original data packets containing 256 bytes will be uploaded to the cloud server. It will be translated into numerical data in the cloud server immediately. Any authorized user can visit it through the UI interface.

**Figure 20:**
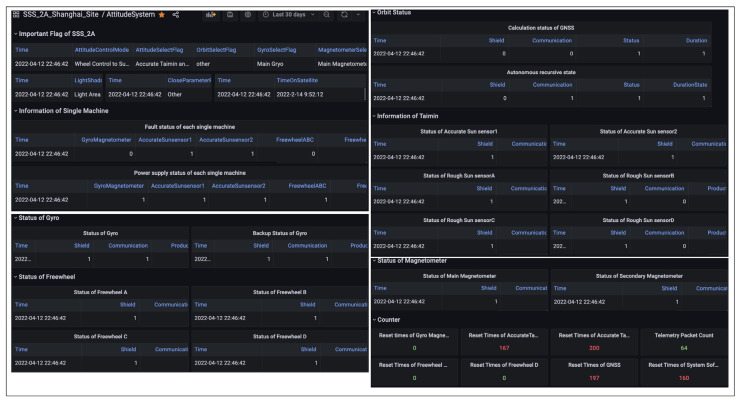
SSS-2A attitude control system data.

**Figure 21:**
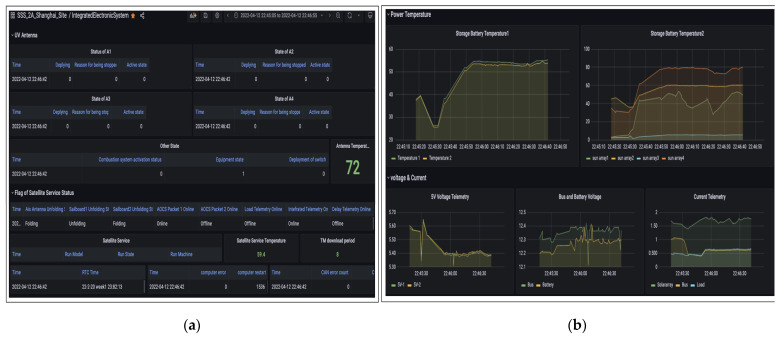
(**a**) SSS-2A integrated electronic system date; (**b**) SSS-2A power system data.

As shown in Figure 20, the data packets of the attitude control system can fully indicate the orbit state and attitude state of SSS-A. All attitude control packets will be stored in the cloud server. For multiple satellites and multiple ground stations, the data packets from different ground nodes or satellites will be stored separately. The data of the integrated electronic system are shown in Figure 21a, which can also be visited on demand. As shown in Figure 21b, in order to clearly indicate the satellite statues, the data of the power system are given in the form of curves. Voltage, current and temperature are key parameters of the power system. The operational life of a satellite can be predicted by these parameters.

Our ground station system satisfies the low power consumption requirements, and the total power consumption of the SDR and Raspberry Pi is less than 5 W. The average power consumption of the antenna system is also less than 5 W.

### 8.2. Networked Service Experiments

The reception performance can be increased by combining multiple ground nodes. As shown in Table 1, there are 18 opportunities for data reception in 24 h. Different numbers of data packets will be received at each link opportunity according to different link states.

As shown in Table 7, a data reception schedule can be constructed by the collaboration strategy. According to our analysis, link duration and pitch angle affect the reception capability severely. However, sometimes, these factors will compromise each other. Up to 2800 packets can be received in 24 h. This result is far beyond the data transmission capacity of the ordinary ground station.

Networked ground stations can also improve remote control capabilities of satellites. If some failures are found when the previous connection is about to be terminated, an early warning can be issued to the cloud server. It will be solved at the beginning of the next connection. Such collaborative working methods could greatly improve the reliability of satellites.

In this section, we presented experimental results of the networked ground station system. The superiority of this design is demonstrated by the data reception capability and plenty of connection opportunities.

## 9. Conclusions

In this paper, a cloud-computing-based networked ground station for microsatellites is introduced to complete the SSS-2A satellite mission. Through orbit and topology analysis, distributing six ground nodes across the country is reasonable. They should collaborate through the network to increase communication efficiency. A cloud server based on Aliyun can deploy a large number of algorithms and control protocols. The operation control is determined by the scheduling algorithm. They are embedded in the cloud server for high operation efficiency. Similarly, the algorithms of adaptive communication are also embedded in the cloud server.

After giving the complete system design, we verified it through on-orbit experiments. As shown in the results, 18 connections can be established, and 2800 data packets can be received in 24 h. This superior performance has surpassed traditional ground stations. Other follow-up work can focus on big-data-based satellite performance prediction.

Additional verification work will continue. Other underdeveloped small satellites will continue to use this system. Quantum communication satellites, gravitational wave detection satellites and amateur radio satellites are all our projects under development. In the future, more satellites and more data will place higher demands on our ground station system.

## Figures and Tables

**Table 1:** System requirements analysis. (√ means meeting the requirements, ○ means not).

Item	Link Duration	Attenuation Fluctuations	Miniaturization	Low Power Consumption
Requirements	√	√	√	√
[11]	○	○	√	○
[12,13]	○	○	√	○
[14]	○	○	√	○
[16]	√	○	○	○
[18]	○	√	○	○
[19]	○	○	○	○
[21]	√	○	○	○
[22]	○	○	○	○
[23]	○	○	○	√
[24]	○	○	○	√

**Table 2:** Link status in 24 h.

Link Num	Start	Over	Duration (s)	Regions	Highest Pitch Angle (°)	Shortest Distance (KM)
1	09:07:57	09:14:16	379	Haerbin	24.5	1097
2	09:13:00	09:17:28	268	Shanghai	15.0	1456
3	10:41:54	10:48:14	380	Haerbin	25.4	1073
4	10:46:06	10:52:14	358	Shanghai	23.1	1108
5	10:45:28	10:52:46	438	Xi’an	46.1	701
6	10:48:04	10:54:42	398	Yunnan	28.8	969
7	12:19:27	12:25:48	381	Xinjiang	25.0	1077
8	12:20:18	12:27:21	442	Qinghai	36.7	821
9	12:24:04	12:26:00	116	Yunnan	10.8	1695
10	13:53:41	13:59:16	335	Xinjiang	19.7	1255
11	19:50:24	19:55:22	296	Haerbin	16.4	1405
12	21:19:16	21:26:43	446	Shanghai	56.6	618
13	21:23:00	21:30:13	433	Haerbin	37.3	820
14	21:23:01	21:26:17	195	Xian	12.3	1615
15	22:52:40	23:00:13	453	Yunnan	76.6	536
16	22:54:51	23:01:46	414	Xi’an	32.2	902
17	22:55:03	23:02:12	428	Qinghai	39.0	791
18	23:00:20	23:02:29	129	Xinjiang	10.9	1700

**Table 3:** Software deployed on cloud server.

Num	Item	Application
1	Cloud server	Ubuntu 20
2	Database 1	MySQL
3	Database 2	MySQL
4	Database 3	MySQL
5	User interface	Grafana
6	Data translation	Python
7	Data forward	UDP

**Table 4:** Warm-up span.

Item	SDR	Power Amplifier	LNA	SoC	Antenna Rotation
span	30 min	10 min	20 min	15 min	5 min

**Table 5:** Basic configure of Raspberry Pi 4.

Item	System	SDR Driver	SDR Software	Interface Script	Cloud Script	Database
Config	Ubuntu 20.04	UHD3.8	Gnu Radio 3.8	python	python	SQL

**Table 6:** Amplifiers parameters.

Item	Gain	P-1	NF	Efficiency	Ports	Voltage
LNA	40 dB	10 dBm	0.5	/	N-J	5 V
PA	25 dB	40 dBm	/	50%	N-J	12.5 V

**Table 7:** Reception schedule in a day.

Link Num	Duration (s)	Regions	Pitch Angle (°)	Received Packets
1	379	Haerbin	24.5	160
2	268	Shanghai	15.0	90
3	380	Haerbin	25.4	163
4	358	Shanghai	23.1	152
5	438	Xi’an	46.1	210
6	398	Yunnan	28.8	177
7	381	Xinjiang	25.0	172
8	442	Qinghai	36.7	205
9	116	Yunnan	10.8	51
10	335	Xinjiang	19.7	143
11	296	Haerbin	16.4	140
12	446	Shanghai	56.6	214
13	433	Haerbin	37.3	211
14	195	Xi’an	12.3	60
15	453	Yunnan	76.6	220
16	414	Xi’an	32.2	201
17	428	Qinghai	39.0	203
18	129	Xinjiang	10.9	30
